# Ergogenic Effects of Combined Caffeine Supplementation and Motivational Music on Anaerobic Performance in Female Handball Players: A Randomized Double-Blind Controlled Trial

**DOI:** 10.3390/nu17101613

**Published:** 2025-05-08

**Authors:** Houda Bougrine, Thierry Paillard, Nidhal Jebabli, Halil İbrahim Ceylan, Julien Maitre, Ismail Dergaa, Valentina Stefanica, Abderraouf Ben Abderrahman

**Affiliations:** 1 Movement, Balance, Performance, and Health Laboratory (MEPS), University Pau and Pays de l’Adour, E2S, 6500 Tarbes, France; houdabougrine@live.fr (H.B.); thierry.paillard@univ-pau.fr (T.P.); maitre.julien@univ-pau.fr (J.M.); 2 Physical Activity Research Unit, Sport and Health (UR18JS01), National Observatory of Sports, Tunis 1003, Tunisia; phd.dergaa@gmail.com; 3 High Institute of Sport and Physical Education of Gafsa, University of Gafsa, Gafsa 2100, Tunisia; 4 High Institute of Sport and Physical Education of El Kef, University of Jendouba, Jendouba 7100, Tunisia; jnidhal@gmail.com; 5 Physical Education of Sports Teaching Department, Faculty of Sports Sciences, Atatürk University, Erzurum 25240, Türkiye; 6 High Institute of Sport and Physical Education of Ksar Said, University of Manouba, Mannouba 2010, Tunisia; abderraouf.benabderrahman@issep.uma.tn; 7 Department of Physical Education and Sport, Faculty of Sciences, Physical Education and Informatics, National University of Science and Technology Politehnica Bucharest, Pitesti University Center, Pitesti 060042, Romania

**Keywords:** caffeine, self-selected motivational music, anaerobic performance, handball, warm-up, repeated-sprint ability

## Abstract

Listening to self-selected motivational music (SSMM) during warm-ups and caffeine (CAF) intake prior to exercise can independently enhance athletic performance among female athletes. Likewise, the potential synergistic effects of these interventions have not yet been thoroughly examined. Objective: The purpose of the study was to assess the independent and combined effects of SSMM during warm-up and pre-exercise CAF intake on maximal short-duration performance in female athletes. Methods: Seventeen female handball players (aged 16.7 ± 0.4 years) participated in a randomized, double-blind, crossover study. Each athlete completed four conditions: (i) placebo (PLA) with no interventions, (ii) music and placebo (MUS), (iii) caffeine intake only (CAF), and (iv) a combination of music and caffeine (MUS + CAF). Performance assessments included the countermovement jump (CMJ), modified agility *t*-test (MAT), repeated-sprint ability (RSA) test (mean and peak sprint performance), and rating of perceived exertion (RPE). Results: The MUS (*p* > 0.05; *p* < 0.01; *p* < 0.01; *p* < 0.001, respectively), CAF (all *p* < 0.001), and MUS + CAF (all *p* < 0.01) conditions significantly outperformed the PLA condition in CMJ, MAT, RSA mean, and RSA peak measures. No significant differences were observed between the CAF and MUS + CAF conditions; however, the best performances were recorded during MUS + CAF. RPE scores remained consistent across conditions. Conclusions: Warm-up routines incorporating either SSMM or a moderate dose of CAF (6 mg·kg^−1^) enhance anaerobic performance in female athletes. While both interventions are effective independently, CAF intake elicits a stronger effect. Although no significant difference was demonstrated for this combination, the concurrent use of SSMM and CAF appears to produce a potential effect, emerging as the most effective strategy for optimizing anaerobic performance.

## 1. Introduction

Athletic performance optimization drives innovation in sports science research [1]. Growing competitiveness in professional sports motivates athletes and coaches to adopt evidence-based enhancement strategies [2,3] to maintain their neurophysiological, psychological, and physical responses [4,5]. Anaerobic capacity, vital in team sports, has been targeted through psychological interventions, nutritional strategies, and specialized training protocols [6,7]. Pre-competition interventions represent a critical research focus, aiming to optimize physiological and psychological readiness immediately before competition.

Among these interventions, caffeine (1,3,7-trimethylxanthine) is widely used as a central nervous system stimulant in sports, with documented performance-enhancing properties across numerous athletic disciplines [8,9,10,11]. Its ergogenic effects block adenosine receptors, facilitate neurotransmitter release, reduce perceived exertion, and improve muscle contractile function [12,13]. Additionally, caffeine is an effective stimulant of central nervous system function. It enhances alertness and wakefulness and reduces drowsiness by blocking adenosine receptors, decreasing the perception of fatigue, and improving alertness and cognition [14].

Evidence supports caffeine dosages of 3 to 6 mg·kg^−1^ administered 60 min before exercise in team-sport athletes [15,16,17], with moderate doses (5–6 mg·kg^−1^) enhancing anaerobic performance in handball and volleyball players [18,19,20]. While caffeine intake has been shown to significantly improve physical performance among female team sports such as basketball [21,22], volleyball [20,23], and handball [19,24,25], emerging evidence suggests its efficacy may be attenuated in female athletes [26]. This reduced effectiveness could potentially stem from sex-specific factors, including differences in body composition, hormonal status, and metabolic processes [27].

Similarly, music serves as an established ergogenic aid in sports performance. Synchronizing music tempo with movement patterns enhances muscle efficiency [28], increases neuronal activity [29], and improves mood [30], attention [31], and self-efficacy [32], leading to performance improvements (oxygen consumption and time to exhaustion) [33,34]. While recent meta-analyses suggest that listening to music has no significant effect on RPE scores [35] or maximal short-term exercise [36,37,38], other studies demonstrate its potential to enhance anaerobic performance [39,40,41]. Self-selected motivational music during warm-up improves power output during high-intensity exercise [42] and enhances team-sport metrics [43], with pronounced effects during morning sessions, when baseline motivation may be lower [43,44].

As presented above, both music and caffeine influence central arousal, but in different ways. CAF directly increases physiological arousal in the central nervous system [14], while music influences psychological states for optimal arousal levels [29].

The combined effects of these interventions remain unexplored in team ball sports like handball, which present distinct physiological demands compared to previously studied combat sports. Numerous meta-analyses have demonstrated that anaerobic performance can be independently enhanced by CAF supplementation [15,45,46,47,48] or listening to music [49,50]. However, only three studies have examined the combined impact of CAF and music on exercise performance [51,52,53]. For instance, Qiu et al. [50] investigated the effects of combining listening to motivational music during warm-up routines with a low CAF dose (approximately 3 mg·kg^−1^) on maximal short-term performance among physically active males. Their findings revealed that this combination improved physical output and enhanced pre-exercise mood and perceived readiness. Similarly, Delleli et al. [51] explored the individual and combined effects of CAF and music on taekwondo-specific performance tasks. They reported that incorporating warm-up music alongside a low CAF dose (3 mg·kg^−1^) significantly improved agility and performance in the 10 s frequency speed-of-kick test among male taekwondo athletes. These combined effects exceeded those achieved by either CAF or music alone and improved psychological outcomes, such as sensory ratings and reduced perceived exertion. In another study, Delleli et al. [52] investigated using warm-up music combined with the same CAF dose (3 mg·kg^−1^) during simulated combat scenarios in female taekwondo athletes. The findings corroborated previous research, demonstrating that the combined intervention improved physiological and psychological outcomes to a greater extent than isolated applications. Female athletes continue to be underrepresented in performance research, despite evidence of sex-specific responses to ergogenic aids. Elite female athletes report higher competition stress levels [54], while physiological differences highlight the need for female-specific research [55,56]. Indeed, despite increased female participation in competitive sports, the development of evidence-based, sex-specific enhancement protocols remains limited [57].

Based on these research gaps, particularly the need for female-specific studies and the unexplored potential of combined ergogenic interventions in team sports, our investigation aimed to assess the independent and combined effects of CAF supplementation (6 mg·kg^−1^) and self-selected motivational music during warm-up on anaerobic performance in female handball players. Crucially, while these interventions have been studied individually, their synergistic effects in female team-sport athletes remain unexplored, making this the first investigation of this performance-enhancing combination in this athlete population. Based on relevant literature data [51,52,53], we hypothesized that a combined approach would produce synergistic effects, resulting in greater performance improvements than either intervention alone.

## 2. Materials and Methods

### 2.1. Sample Size Calculation

Following the procedures outlined by Beck [58], G*Power software (version 3.1.9.6; Kiel University, Kiel, Germany) was employed to calculate the required sample size before conducting the study [59]. A significance level (α) of 0.05 and a statistical power (β) of 0.95 were established. Based on the findings of Delleli et al. [52], who reported a moderate effect of caffeine ingestion on agility in female players and further deliberation among the authors, an estimated effect size of f = 0.4 was adopted. According to this estimation, a minimum sample size of 15 participants was needed to ensure the desired statistical power and mitigate the risk of type II error.

### 2.2. Ethics Approval

The study protocol adhered to the ethical principles of the Declaration of Helsinki for research involving human subjects and received approval from the local research ethics committee of the High Institute of Sport and Physical Education of El Kef, University of Jendouba, Jendouba, Tunisia (reference PH-077/2023). It also complied with the ethical and procedural requirements for the conduct of sports medicine and exercise science research [60]. Since participants were minors, written informed consent was obtained from their parents or legal guardians before participation, along with verbal assent from the participants. This consent process followed a thorough explanation of the study’s methodology and a discussion of its potential risks and benefits.

### 2.3. Participants

Of the 34 female handball athletes screened, 24 met the inclusion criteria. Ultimately, only 17 participants completed the full study protocol and were included in the final analysis. One athlete withdrew, and 6 participants were excluded due to issues related to their menstrual cycles.

The selected 17 participants (mean ± SD: age 16.76 ± 0.4 years; height 1.64 ± 0.1 m; body mass 59.43 ± 6 kg; BMI 21.96 ± 1.8 kg/m^2^) met the following eligibility criteria: (a) aged 15 to 18 years; (b) daily caffeine intake below 0.99 mg·kg^−1^·day^−1^ (classified as low caffeine consumers) or higher than 25 mg/day (not caffeine-naïve) [61]; (c) a minimum of 3 years of active participation in team ball sports, with at least three sessions per week for the last 6 months; (d) normal weight based on body mass index (18 to 23 kg/m^2^), (e) an absence of medications, dietary supplements, or performance-enhancing substances that could influence the study outcomes; (f) regular menstrual cycles with no more than a 3-day variation over the last 4 months [62]; and (g) consistent sleep patterns.

Exclusion criteria included: (a) extreme chronotypes; (b) a positive history of alcohol or tobacco use; (c) potential caffeine allergy; (d) a daily caffeine consumption below 25 mg/day (defined as caffeine-naïve); (e) a history of menstrual irregularities within the past 4 months or the use of oral contraceptives or related medications in the previous 4 months (including pills, patches, injections, implants, or intrauterine devices); (f) any chronic medical conditions or the use of medications to manage such conditions; and (g) any diseases and abnormalities of the ear or hearing.

Each athlete had at least 3 years of competitive experience in handball (mean ± SD experience of 4.9 ± 0.7 years) and had maintained regular engagement with a minimum of 3 training/competition sessions per week (mean 4.8 ± 0.4 sessions/week) over the preceding 6 months. Participants were identified as low caffeine consumers with an average daily intake of 43.55 ± 10.67 mg/day (0.73 ± 0.15 mg·kg^−1^·day^−1^), adhering to the recent classification previously defined as low caffeine consumers [61], with a range of daily consumption between 25 mg/day and 0.99 mg·kg^−1^·day^−1^.

While previous studies [63,64] revealed no effect of the menstrual cycle phase on CAF or warm-up music efficacy, all menstrual cycle lengths (28 ± 1.9 days) and phases were evaluated using the My Calendar^®^ Period Tracker [65]. Under established methodologies, assessments were conducted only throughout the follicular and luteal phases [19,24].

### 2.4. Experimental Protocol

**Figure 1:**
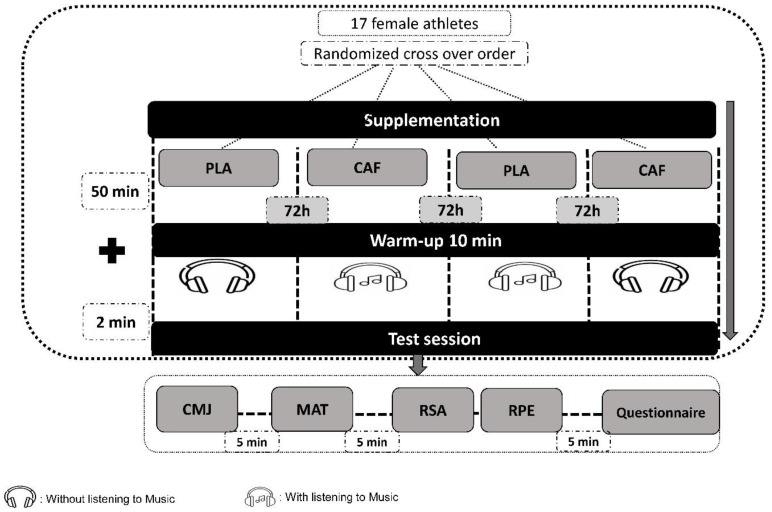
Experimental design. PLA: placebo (cellulose), CMJ: countermovement jump, MAT: modified agility *t*-test, RSA: repeated-sprint ability, RPE: rating of perceived exertion; CAF: 6 mg.kg^−1^ CAF intake.

A double-blind, randomized, placebo-controlled crossover methodological approach was employed in this study, where each participant served as their own control to examine the acute effects of CAF intake combined with listening to music during warm-up on the anaerobic performance in female players. Randomization was applied using an online software tool (https://randomizer.org/#randomize, accessed on 4 October 2023). The participants completed four distinct experimental sessions, with a minimum washout period of 72 h between sessions, ensuring full recovery and clearance of substances from the body [19]. During these trials, the participants were exposed to one of four conditions: (i) placebo (PLA) with no interventions, (ii) music and placebo (MUS), (iii) CAF intake only, and (iv) a combination of music and CAF intake (MUS + CAF). CAF and placebo supplementation were administered fifty minutes before the warm-up protocol. Randomization, blinding, and preparation of the supplements were conducted by an independent investigator who was not involved in the data collection process. Athletes completed a standardized warm-up lasting approximately 10 min, which commenced 50 min after CAF administration across the four testing trials. The testing order remained consistent across all sessions, starting with the countermovement jump (CMJ) test, then proceeding to the modified agility *t*-test (MAT), repeated-sprint ability (RSA) test, rating of perceived effort (RPE) test, and concluding with the CAF side effect questionnaire [66]. Moreover, a five-minute rest period was provided between each test to allow for sufficient recovery (Figure 1).

#### 2.4.1. Warm-Up Protocol

Fifty minutes after consuming the capsules, athletes completed two warm-up protocols. The warm-up routine consisted of 3 min of jogging (at a pace of 8–10 km/h) and three minutes of dynamic stretching exercises targeting the whole body. This was followed by a three-minute session incorporating sprinting drills, such as ankle kicks, high knees, back kicks, and skipping, and an additional two minutes of sprinting. Participants performed 10 min (including rest) of running and executing without music (PLA and CAF conditions) or performing the same warm-up while listening to their self-selected music for motivation (MUS and MUS + CAF conditions). Following both protocols, athletes rested for two minutes.

#### 2.4.2. Music Protocol

Music self-selection occurred before the familiarization and testing sessions [52,53,67,68]. The researchers documented each participant’s chosen music selection during the familiarization session. They compiled a customized playlist, labeled with the participant’s name, to be used during the warm-up phase of the experimental trials. The music selection for each participant was self-chosen and evaluated as motivating using the Brunel Music Rating Inventory-2 [69] to quantify the motivational impact of their choice. For use during the music conditions, the tracks were required to have a tempo of at least 120 bpm to ensure a stimulatory effect [70], with the average tempo of the chosen music reported as 139.5 ± 17.3 bpm. Participants listened to the music through headphones connected to their mobile devices, with a single track being used consistently across all sessions. The volume was standardized at 80 dB for all participants and verified using the decibel sound level meter application (developed by Vlad Polyanskiy). During music conditions, the selected tracks were played throughout the warm-up, with looping enabled if a track’s duration was less than the 10 min warm-up period, being repeated to ensure continuous playback. Furthermore, athletes wore headphones for the conditions without music, but no music was played, ensuring uniform testing conditions.

#### 2.4.3. CAF Administration Protocol

Two hours before the start of the exercise protocol, participants arrived at the indoor court for the experimental trials. To further minimize digestive discomfort and maintain consistency with fasting guidelines, participants were served a controlled breakfast consisting of around 500 kcal, consumed two hours before the trials, following methods outlined in previous studies on pre-exercise CAF intake [19,24], diverging from their habitual pretrial dietary practices. During all conditions, participants ingested capsules containing either a placebo made of a non-active substance (cellulose; Guinama 6, Valencia, Spain) or 6 mg·kg^−1^ of CAF (Bulk Powders, Colchester, UK). Participants were instructed to stay seated and rest for 50 min following capsule consumption. These capsules were administered following a double-blind methodology to ensure the reliability of the study’s outcomes. The CAF dose was selected based on its proven effectiveness in enhancing young female handball performance while causing minimal to no adverse effects in this population [18,19,24]. To promote optimal absorption, the capsules were consumed with 100 mL of water exactly one hour before the experimental trials, with participants adhering to a fasting period of at least 60 min after their last meal. The 60 min timing for consumption was selected because CAF achieves maximum plasma concentration approximately one hour after ingestion, facilitated by its rapid absorption in the gastrointestinal tract [71].

#### 2.4.4. Blinding Protocol

To maintain blinding integrity, all capsules were identical in appearance and were prepared by a research team member who was not involved in the investigation, and participants were instructed not to discuss or compare the taste of the supplements. Compliance with consumption was monitored by the investigators and the athletes’ coaches. To further assess the effectiveness of blinding, participants were verbally asked to identify the treatment after consumption. Importantly, participants largely failed to identify the interventions, with none correctly discerning all four conditions, confirming successful maintenance of blinding and supporting study integrity.

#### 2.4.5. Familiarization Procedure

Over the four weeks before the experiment, participants visited the facility multiple times for music selection, preliminary data collection, and familiarization sessions to minimize learning effects. During the first visit, the researchers explained the study’s procedures, obtained informed consent, and collected anthropometric data. At the same time, participants completed various questionnaires, including those on daily caloric intake, the Pittsburgh Sleep Quality Index (PSQI) with the validated Arabic version [72], the validated Arabic version of the Horne and Östberg questionnaire [73], and habitual CAF consumption [74]. Participants’ body weight was measured using the Tanita BC-545n digital scale (Tanita Corporation, Arlington Heights, IL, USA) with a precision of 0.1 kg, ensuring minimal clothing, bare feet, and a fasted state. After establishing the optimal CAF dosage for each individual, athletes were asked to record their food intake before the initial session and to keep their diet unchanged for the following sessions. To reduce learning effects and maintain result consistency, all participants underwent practice sessions at 9:00 AM, the same time as the actual trials during the two visits before testing. This allowed them to become familiarized with the procedures and equipment.

#### 2.4.6. Standardization and Control Procedures

Participants were instructed to abstain from CAF consumption and strenuous physical activity for 24 h before each trial. A detailed list of foods and beverages containing CAF was provided, and participants were advised to avoid these items throughout the testing day and the 24 h leading up to each trial session. To ensure consistency, participants documented their activities during the 24 h before the initial trial and were required to replicate this routine before the subsequent trials. These activity records were photocopied and distributed to participants for reference, enabling uniform adherence to dietary and activity guidelines across all sessions. Before the start of each session, participants verbally confirmed compliance with the dietary restrictions and affirmed they had avoided all prohibited substances containing CAF. Additionally, participants maintained their habits regarding lifestyle, training, sleep, hydration, and diet throughout the study, ensuring the same routine preparation was followed 24 h before each trial. All participants abstained from using screens (phones, tablets, computers, televisions) for 24 h before testing sessions to avoid any impact of blue light on their sleep quality and subsequent performance [75,76,77,78]. Body mass was recorded consistently during the morning sessions throughout the various familiarization periods to ensure accurate measurements for calculating the appropriate CAF dosage trials. To ensure consistency throughout the study, all testing sessions were conducted simultaneously under identical conditions (9:00 AM) [75,76,77]. The same indoor court was utilized, and equipment and testing sequences remained consistent under the continuous supervision of the research team. Environmental factors, such as an ambient temperature of approximately 28 °C and relative humidity of 46%, were maintained steadily across all four experimental sessions.

Furthermore, participants began testing at 9:00 AM following the sequence: CMJ → 5 min rest → MAT → 5 min rest → RSA (with post-sprint RPE). To maintain standardization while optimizing efficiency, we used duplicate stations (two Optojump systems, MAT circuits, and RSA setups), and athletes were randomly divided (independent of study condition randomization) into two groups: group A (Optojump A→MAT A→RSA A) and group B (Optojump B→MAT B→RSA B). Experimenters recorded completion times to enforce 5 min rest periods, maintaining standardization while enabling parallel testing.

#### 2.4.7. Assessment of Habitual Consumption and Adverse Effects Related to CAF Intake

An adapted version of a food frequency questionnaire (FFQ), as recommended by Bühler et al. [74], was employed to evaluate the habitual CAF intake of participants. The assessment of daily food consumption for the month preceding the study was conducted individually using household measurements, consistent with prior guidelines [61]. A qualified nutritionist referenced nutritional tables to calculate the athletes’ daily CAF intake during the four weeks before the experimental phase, considering the total CAF consumption and the participants’ body mass, as Filip et al. [61] advised. To maintain uniformity within the sample and mitigate classification inconsistencies that could impact the analysis, only athletes classified as low CAF consumers, with a daily CAF intake ranging from 25 mg/day to 0.99 mg·kg^−1^·day^−1^, were included in the study.

To examine possible adverse effects such as headaches, disturbances in sleep, or gastrointestinal discomfort associated with CAF use, participants were asked to complete a nine-item questionnaire. This instrument utilized a yes/no response format, in line with the framework proposed by [66]. The questionnaire was administered immediately after each experimental session (day 1) and again 24 h later (day 2) to capture any delayed or continuing side effects.

### 2.5. Outcome Measures

#### 2.5.1. Countermovement Jump (CMJ) Test

Participants performed a vertical jump, beginning with a downward preparatory motion followed by an explosive upward phase while keeping their torsos upright. Each participant executed three jumps, separated by two-minute rest intervals [19,79,80,81], and was instructed to maximize jump height while landing in the same starting position, using arm swings for momentum. Jump height (in centimeters) was recorded using the Optojump Next system (Bolzano, Italy) and Microgate software (v.1.13.24; Optojump software v.1.10.50), with only the for the jump’s height data retained for analysis.

#### 2.5.2. Modified Agility *t*-Test (MAT)

Participants’ agility was assessed using a modified *t*-test, featuring four cones arranged in a “T” design [82]. The test incorporated multidirectional movements, including forward sprints, lateral shuffles, and backward pedaling. Performance was timed using the Witty photocell system (Microgate^®^, Bolzano, Italy), with participants starting 0.5 m behind the initial timing gate. The best performance was determined by recording the fastest completion time out of two trials, with a three-minute recovery period between attempts.

#### 2.5.3. Repeated-Sprint Ability Test (RSA Test)

Participants completed six maximal-effort shuttle sprints (2 × 12.5 m per sprint) with 20 s passive recovery intervals between efforts. Each sprint required 180° turns, simulating the multidirectional demands of team ball sports for female athletes [19,24,83]. Sprint times were recorded using Witty timing gates (Microgate^®^, Italy), with participants starting 0.5 m behind the start line after a 3 s visual countdown from a Microgate^®^ light panel. The digital timer automatically triggered when the photocell beam was broken, and athletes were instructed to sprint 12.5 m, touch the line with their feet, and immediately return at maximal effort while receiving standardized verbal encouragement throughout all trials. Key performance metrics included the best sprint time (RSA peak) and the mean sprint time across all trials (RSA mean), which were recorded for further analysis.

#### 2.5.4. Rating of Perceived Effort (RPE)

Participants shared how hard they felt they were working after every 25 m shuttle in the RSA test. Their responses were recorded using the Borg scale, which ranges from 0 (total rest, like sitting in a chair) to 10 (maximum effort and strenuous activity) [84]. These effort ratings were collected immediately after each sprint. An average was then calculated from all six sprint scores to represent the overall effort level for that session. This method helped to ensure consistency when analyzing effort levels during the test.

### 2.6. Statistical Analysis

All statistical analyses were conducted using STATISTICA 10 software (StatSoft, Paris, France). The mean and standard deviation (SD) were calculated for each variable. Data normality was confirmed through the Shapiro–Wilk test. The influence of the CAF supplementation and/or music was evaluated using a one-way repeated-measure ANOVA (4 Conditions). When significant differences between means were identified, the Bonferroni post hoc test was applied for further analysis. Effect sizes were calculated using the partial eta-squared statistic (ηp2), with thresholds defined as 0.01 for a small effect, 0.06 for a moderate effect, and 0.14 for a large effect, based on Cohen’s criteria [85]. Standardized effect size (Cohen’s d) was employed to assess the magnitude of differences between variables, as categorized by prior research [86]: trivial (d ≤ 0.20), small (0.20 < d ≤ 0.60), moderate (0.60 < d ≤ 1.20), large (1.20 < d ≤ 2.0), very large (2.0 < d ≤ 4.0), and extremely large (d > 4.0). The significance threshold was set at *p* ≤ 0.05. GraphPad Prism 8 (GraphPad Software, San Diego, CA, USA) was used to generate all results.

## 3. Results

### 3.1. CMJ

**Figure 2:**
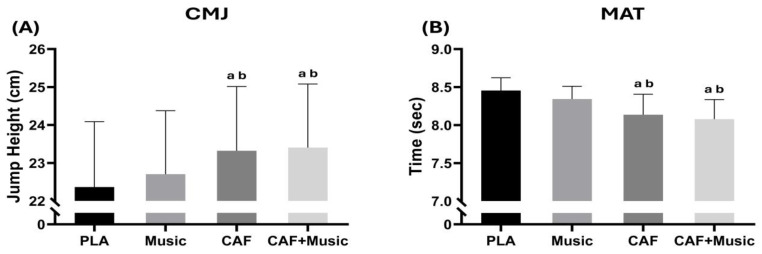
Mean ± SD values of the countermovement jump (CMJ) test (**A**) and modified agility *t*-test (MAT) (**B**) recorded under four conditions: PLA, placebo; music, listening to motivational music without CAF intake; CAF, 6 mg.kg^−1^ CAF intake without listening to motivational music; CAF + music, 6 mg.kg^−1^ CAF intake with listening to motivational music. a: Significant difference compared to the PLA condition (*p* < 0.05); b: significant difference compared to the music condition (*p* < 0.05).

There was a moderately significant main effect of conditions [F (3–48) = 31.22; *p* < 0.001; η^2^p = 0.66] for CMJ height. The Bonferroni test indicated better performance for the CAF (+4.3%, *p* < 0.001) and CAF + MUS (+4.7%, *p* < 0.001) conditions than for the PLA condition. Additionally, the CAF (*p* < 0.001) and CAF + MUS (*p* < 0.001) conditions were significantly different from the MUS condition (Figure 2).

### 3.2. Modified Agility t-Test (MAT)

The one-way ANOVA revealed a small, but significant main effect of conditions [F (3–48) = 19.69; *p* < 0.001; η^2^p = 0.55] on MAT performance. The CAF (−3.8%, *p* < 0.001) and CAF + MUS (−4.3%, *p* < 0.001) conditions reduced run times more than the PLA condition. A significant difference between the CAF and MUS conditions was found (*p* < 0.01). Furthermore, there was a significant difference between the MUS condition and the CAF + MUS (*p* < 0.001) condition (Figure 2).

### 3.3. RSA

**Figure 3:**
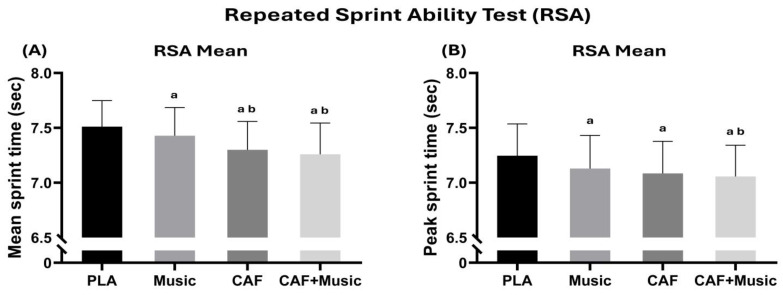
Mean ± SD values of the RSA mean (**A**) and RSA peak (**B**) recorded under four conditions: PLA, placebo; music, listening to motivational music without CAF intake; CAF, 6 mg·kg^−1^ CAF intake without listening to motivational music; CAF + music, 6 mg.kg^−1^ CAF intake with listening to motivational music. a: Significant difference compared to the PLA condition (*p* < 0.05); b: significant difference compared to the music condition (*p* < 0.05).

There was a moderately significant effect on RSA mean performance [F (3–48) = 97.04; *p* < 0.001; η^2^p = 0.85]. The analysis of post hoc tests revealed that RSA mean times significantly decreased when comparing the PLA condition with the MUS (−1.4%, *p* < 0.01), CAF (−2.8%, *p* < 0.001), and CAF + MUS (−3.4%, *p* < 0.001) conditions. The CAF (*p* < 0.001) and CAF + MUS (*p* < 0.001) conditions were different from the MUS condition (Figure 3).

There was a moderately significant main effect of conditions on RSA peak [F (3–48) = 43.17; *p* < 0.001; η^2^p = 0.72]. The MUS (−1.6%, *p* < 0.001), CAF (−2.2%, *p* < 0.001), and CAF + MUS (−2.6%, *p* < 0.001) conditions were different from the PLA condition. There was no significant difference between the MUS and CAF + MUS (*p* > 0.05) and CAF conditions (Figure 3).

### 3.4. Rating of Perceived Effort (RPE)

**Figure 4:**
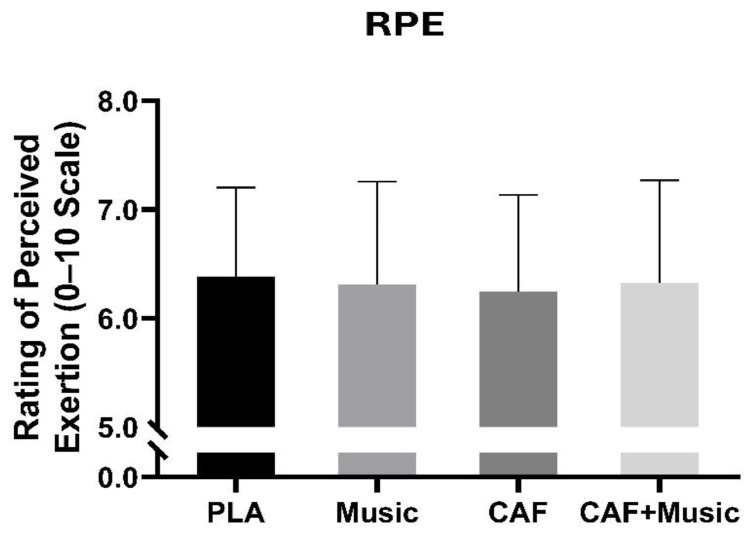
Mean ± SD values of rating of perceived exertion (RPE) recorded under four conditions: PLA, placebo; Music, listening to motivational music without CAF intake; CAF, 6 mg·kg^−1^ CAF intake without listening to motivational music; CAF + Music, 6 mg.kg^−1^ CAF intake with listening to motivational music. a: significant difference compared to the PLA condition (*p* < 0.05); b: significant difference compared to the Music condition (*p* < 0.05).

No significant main effect conditions were detected for the RPE scores [F (3–48) = 0.73; *p* = 0.53; η^2^p = 0.04], indicating that the RPE scores remained unchanged across all conditions (Figure 4).

### 3.5. CAF Side Effects

The occurrence of side effects from the CAF ingestion was relatively low, ranging from none to 17.64% (3 participants from 17) during both the PLA and MUS trials on the same day (day 1) and the following day (day 2).

Minor side effects were observed after the administration of the CAF, with reports also ranging from none to (17.64%; 3/17 participants) on both days. Specifically, on the same-day test, there were increased reports of tachycardia (11.67%; 2/17 participants), headaches (11.67%; 2/17 participants), and gastrointestinal issues (11.67%; 2/17 participants). When CAF was taken alongside a warm-up routine paired with self-selected motivational music, adverse symptoms were consistent across both the same and the following day, ranging between none and 23.52% (4/17 participants). On the same-day test, heightened reports of headaches (11.67%; 2/17 participants) were documented, along with increased vigor or activeness (23.52%; 4/17 participants) and a perceived improvement in performance (23.52%; 4/17 participants). Minor side effects of tachycardia and headaches (both 5.88%; 1/17 participants) were reported. The following day, minor side effects were documented, including gastrointestinal issues (5.88%; 1/17 participants), insomnia (11.72%; 2/17 participants), and increased urine output (5.88%; 1/17 participants). However, none of the participants reported tachycardia or headaches (Table 1).

Notably, athletes struggled to correctly identify pre-exercise CAF intake, with correct identification rates of 23.52% (4/17 participants) for CAF and 17.64% (3/17 participants for the CAF + MUS trial. Only 11.76% (2/17) of participants correctly identified that they had taken a placebo without music, and 23.52% (4/17) of participants identified placebo intake with music. Importantly, none of the athletes could correctly identify all four conditions, indicating that the study’s blinding was successfully maintained (Table 2).

## 4. Discussion

The present study demonstrated that caffeine supplementation and self-selected motivational music during warm-up independently enhanced anaerobic performance in female handball players. Caffeine administration (6 mg·kg^−1^) significantly improved more than music alone across all performance parameters. While the combination of caffeine and music did not outperform caffeine alone significantly, this dual-intervention approach yielded the highest absolute values in countermovement jump height, agility, and repeated-sprint ability. The observed performance improvements occurred without significant changes in perceived exertion or notable side effects, suggesting good tolerability of these interventions in this population.

### 4.1. CAF Intake Effects

Our findings demonstrated that the single ingestion of CAF, alone or combined with listening to MUS, increased CMJ jump capacity by 4.3% during CAF and 4.7% during CAF + MUS compared to the PLA condition. This moderate dose of CAF (6 mg.kg^−1^) was chosen based on its observed impacts on muscle performance during team sports and its proven ergogenic effects on short-term maximal performance in female athletes without causing significant side effects when consumed in the morning [16,19,24]. A meta-analysis by Grgic and Varovic [47] presented CAF as a reliable means of increasing muscle power when assessing vertical jump height in female athletes. Another study conducted on a team of world class volleyball athletes also observed changes in CMJ measures, such as flight time (+5.3%), peak power (+16.2%), and peak concentric force (+6.5%), with no reported side effects when taking CAF at a dose of 5 mg/kg [87]. Previous studies involving young female handball players demonstrated that a moderate CAF dosage of 6 mg.kg^−1^ effectively enhanced their jumping performance [18,19,24,25]. Additionally, it has been recently revealed that 5 mg.kg^−1^ of CAF intake can improve CMJ in female volleyball players [20]. However, other studies have failed to identify a CAF-induced improvement in CMJ [88,89]. These contradictory findings compared to the present study may be related to methodological differences (age [90], the timing of CAF administration and habitual CAF consumption [25,91,92], sex [93], genotype [94,95], ingested doses [24,96], and participant physical level [97].

Regarding agility, our findings showed that agility improved significantly under the CAF condition (−3.8%, *p* < 0.001) and the CAF + MUS condition (−4.3%, *p* < 0.001). CAF is an adenosine antagonist that increases dopaminergic and noradrenergic activity, enhancing reactivity and neuromuscular coordination [98]. The present study results are consistent with previous investigations among female athletes [18,19,24], who reported that CAF intake improves agility in young female handball players. These findings also align with a study conducted by Jebabli et al. [99], who reported that 5 mg·kg^−1^ of CAF significantly improved repeated modified agility test performance among physically active young male and female students. A moderate dose (6 mg·kg^−1^) of CAF did not enhance performance on agility tests among female basketball or volleyball players [100]. Similarly, lower doses of CAF have shown no significant effect on agility in female volleyball [101], basketball [22], or handball players [102].

Additionally, our results revealed that CAF ingestion improved RSA performance (−2.2%), while the CAF + MUS combination improved it (−2.6%). Previous studies have demonstrated that a moderate dose of CAF (6 mg·kg^−1^) can improve the number of repeated sprints and the distance covered by female footballers [100] and RSA mean and peak time among female handball players [19,24], which is also consistent with the present study. However, Buck et al. [103] did not observe significant effects in 6 × 20 m repeated-sprint ability with the same dose of CAF among female team ball players.

Furthermore, RPE scores were unaffected by CAF supplementation, consistent with meta-analyses [17,104] showing minimal improvements in jumping, sprinting, agility, and endurance in team sports with moderate CAF doses (3–6 mg·kg^−1^), and no change in perceived exertion. A recent meta-analysis on women’s team sports [16] further supports this, suggesting that short recovery periods between sprints may not allow CAF to counteract fatigue. These findings indicate that mechanisms other than reduced RPE likely contribute to CAF’s ergogenic effects, such as enhanced muscle fiber conduction and motor unit recruitment [105,106]. Regarding the mechanism stimulated by CAF, it can enhance anaerobic exercise via the stimulation of Ca^2+^ and K^+^ and improve cross-bridge formation by stimulating the release of Ca^2+^ from the sarcoplasmic reticulum, thus causing an enhancement in muscle strength [107]. These increases could result from the effects of CAF on the excitation–contraction coupling to release calcium and activate the Na^+^/K^+^ pump [12]. CAF-stimulated glycolytic metabolism could also explain these positive results, which lead to catecholamine release and enhance ATP supply for immediate exercise [107].

Existing literature on female team-sport athletes, including meta-analysis findings [16], demonstrates that CAF intake significantly enhances physical performance in basketball [21,22], volleyball [20,23], and handball [19,24,25]. These ergogenic effects may be partly mediated by improvements in reaction time and decision-making [18,19,99], critical factors in dynamic team sports like handball and volleyball, where rapid, accurate responses to opponents’ actions determine competitive success. A recent study suggests that neuromodulatory mechanisms, such as orexin A, influence attention and reaction time following neurostimulation in elite female volleyball players, offering a neurophysiological basis for sex-specific performance optimization. These findings suggest that neurocognitive arousal, including mental flexibility, interacts synergistically with ergogenic strategies (e.g., CAF, music, motivational interventions) to enhance athletic performance. Given the shared neurocognitive mechanisms underlying these responses, discipline-specific protocols may be warranted for female team-sport athletes. Additionally, sex-specific factors such as hormonal fluctuations (e.g., estrogen-modulated adenosine receptor sensitivity [16,108] and interindividual variability [109] may further modulate CAF’s psychostimulant effects on stress and motivation [110]. These insights underscore the need for tailored guidelines to optimize women’s team-sport performance.

### 4.2. Effects of Listening to Music 

Regarding the effects of music, while listening to music alone was effective in enhancing RSA parameters, it was not sufficient to improve CMJ or agility. However, when combined with CAF, music proved effective for all these parameters, resulting in higher values than when each condition was applied independently. Although no significant difference was reported between music and CAF, this highlights the potential for combined interventions to yield superior performance outcomes.

Our results corroborate previous findings that listening to preferred music enhances performance through increased cortical activation and reduced neural inhibition [67]. Music demonstrated the ability to improve athletic performance by diverting attention away from feelings of fatigue [111]. Additionally, it can influence psychomotor arousal, acting either as a calming agent or as a source of stimulation, depending on the athlete’s specific needs and situational demands [40,112]. This study supports previous research showing that motivational warm-up music can boost power output and total work during repeated sprints in female collegiate athletes [42]. Nevertheless, the present study is inconsistent with Eliakim et al. [113], who reported that music did not affect RSA performance (total time, ideal time, and fatigue index) during 12 × 20 m bouts in young female basketball players. Additionally, ref. [30] concluded that using self-selected high-tempo, moderate-intensity music at a regular frequency would be an effective ergogenic stimulant. This type of music was shown to improve female athletes’ mood more effectively compared to male athletes during an intermittent kickboxing anaerobic speed test. Music’s rhythmic and affective properties attenuate perceived exertion in female athletes, allowing them to maintain higher physical and cognitive performance [30,63]. This combined effect of music and caffeine on perceived exertion and neuronal activity confirms its effectiveness in improving physical performance. However, future studies should focus more on the interactive effects of caffeine and music to clarify their potential impact on neurocognitive activation between genders.

During warm-ups, motivational music enhances athletic performance, particularly in total distance and high distances covered [43]. A recent systematic review and meta-analysis suggests that listening to music during the Wingate anaerobic test may have a positive physiological impact on relative anaerobic exercise performance. However, the exact mechanisms behind this effect are not yet fully understood [49].

While previous studies [36,37,38] reported no significant effects of motivational music on short-term, high-intensity efforts, other research has demonstrated that incorporating music during warm-ups can significantly boost peak power and overall performance [43,114,115]. Similarly, Jebabli et al. [116] reported that listening to music improves jump height during repeated CMJs to exhaustion. These differences in findings may result from variations in participants’ fitness levels, the type and intensity of effort [117], the type of music employed, or whether the music was used during the exercise itself or solely during the warm-up phase [43,118]. Recent research has highlighted that the timing of music exposure plays an important role, with music during workouts significantly enhancing both emotional well-being and anaerobic performance [119]. The effect of music also seems linked to self-esteem, confidence [120], and time of day, especially in the morning, when motivation is sometimes low. Listening to music during warm-up seems to be a more sufficient strategy in the morning than in the afternoon [39,43,44]. Additionally, studies indicate that synchronizing music tempo to an individual’s movements boosts motor efficiency and optimizes energy expenditure, improving kinetic and physiological results [121].

The RPE scores recorded after the warm-up showed no significant differences between the conditions with and without music. This finding contrasts with the results of studies [43,114,122]. While recent meta-analyses argue that preferred music does not alter perceived exertion [35], it has been revealed that following the 5 m multiple shuttle run test, RPE scores were higher after warm-up without music compared to warm-up with music in the morning [122,123]. However, refs. [42,114,115] failed to demonstrate any impact of listening to music on RPE scores. Such discrepancies among research findings are possibly related to the exerciser’s psychological aspects, including the exercise’s context and the individual’s mental state during performance. Indeed, factors like personality traits, motivation levels, and attentional focus affect RPE scores during physical activity [124].

CAF-related side effects were rare, with only a limited number of instances reporting headaches or tachycardia. This highlights the practicality and safety of incorporating CAF and motivational music into warm-up strategies for young female handball athletes. These findings align with previous research on female team-ball-sport athletes, indicating that consuming a moderate dose of CAF in the morning (the condition of the present investigation) typically minimizes side effects compared to afternoon intake, when such effects tend to be more pronounced [19,24]. The combination of CAF + MUS does not appear to enhance these effects, and music has therefore been shown to affect some physiological responses to CAF.

### 4.3. Combined Effects of CAF + MUS

Although no significant differences were revealed when comparing the MUS + CAF condition to the CAF condition, this led to the highest reported values across all performance parameters compared to the conditions involving only music or only CAF intake. The combined action of CAF and MUS in the present study appeared to amplify the induced benefits of CAF by enabling an optimal activation state. These results suggest that the dual stimulation of physiological and psychological pathways may enhance overall ergogenic effects, providing a more comprehensive and practical performance boost. Several meta-analyses have shown that CAF supplementation or listening to music [48,49] can independently enhance anaerobic performance [15,45,46,47,48]. Likewise, only a few studies have examined the combined effects of CAF and music on exercise performance [51,52,53]. Ref. [53] examined how pairing self-selected music during warm-up sessions with a low dose of CAF (around 3 mg.kg^−1^) impacted anaerobic performance in physically active males. That study found that this combination increased physical performance and improved mood and perceived readiness before exercise. Delleli et al. [52] evaluated the same combination in female taekwondo athletes during simulated combat scenarios, confirming that this approach led to superior physical and psychological outcomes compared to either intervention by itself. Similarly, a recent study analyzed the individual and combined impacts of CAF and music on taekwondo-specific tasks, reporting that warm-up music alongside a low CAF dose (3 mg·kg^−1^) significantly enhanced agility and 10 s frequency speed-of-kick test performance in male taekwondo athletes [51]. These effects were greater than either intervention alone and included psychological benefits, such as improved sensory ratings and reduced perceived exertion. However, other studies have shown that neither CAF consumption nor music listening during warm-up improved repeated-sprint performance [89,116]. Therefore, the dosage of CAF consumption, the type of motivating music and its listening time, as well as the fitness status of the participants (trained or not), are key elements in improving physical performance [96,116].

Contrary to expectations, no difference was recorded in RPE during CAF and MUS conditions. In other words, the unique and combined effect of CAF and MUS improves physical performance without significant changes in RPE value. The stability of RPE may reflect a capacity to dampen afferent neural signals, which could reduce immediate physiological responses, even as physical performance demands increase [125]. These outcomes are consistent with the conclusions of some authors [43,122], which indicated that RPE scores tended to be higher following warm-up sessions conducted without music than those with music. Conversely, previous studies [42,114,115] and recent meta-analyses [35,126] suggest that preferred music does not significantly influence RPE scores. These inconsistencies across various studies may stem from psychological variables tied to the participants, such as the exercise context or their mental state while performing.

Factors influencing CAF’s ergogenic effects are varied and complex. These include genotype [94,95], age [90], regular CAF consumption habits [91], sex [93], and the source and form of CAF [127]. Additional considerations include the length of CAF abstinence before studies, possible side effects [128], the type of physical activity performed [12], the time of day [24], the level of training [129], various doses [19], and psychological influences like the nocebo effect [130], all of which significantly impact its performance-enhancing potential. Despite promising findings, the combined effects of CAF and music on anaerobic performance remain underexplored, with limited studies challenging direct comparison. While CAF and music during warm-ups are known for their ergogenic benefits, their synergistic impact, especially in female athletes and team sports, remains unclear. Thus, the present results indicate that combining a moderate dose of CAF with motivational music during warm-up is an effective and low-budget way to improve performance without generating significant side effects among female handball athletes.

### 4.4. Limitations

This investigation is not without its limitations, which warrant acknowledgment. One notable limitation is the absence of blood CAF-level measurements, leaving uncertainties about the absorption and impact of this moderate dosage. Future research should assess the effects of different music types, tempos, and different states (fatigue or rest state) across diverse sports contexts to deepen our understanding of these variables further. Furthermore, the study did not measure neurochemical variables such as orexin or dopamine, which are joined in hypothesized causal chains to some of the observed performance effects. These neurochemicals partake in arousal, motivation, and cognition, and their accurate measurement would have opened new avenues for examining mechanisms through which caffeine and music exert their ergogenic effects. Another limitation is excluding menstrual cycle influences on performance and CAF-related benefits. Although previous research suggests that female sex hormones might diminish the performance improvements associated with CAF, this interaction was outside the scope of this study and requires further exploration. Participants adhered to a controlled dietary protocol, avoiding CAF-rich foods before and between sessions, with urine samples confirming compliance. However, due to the small sample (*n* = 17) and restricted age range, these findings from young female athletes may not generalize to other populations, including males, older adults, sedentary individuals, or heavy caffeine consumers.

## 5. Conclusions

Our experimental findings showed that self-selected motivational music during warm-up and pre-exercise caffeine supplementation (6 mg·kg^−1^) independently enhanced anaerobic performance measures in female handball players. Caffeine administration elicited more substantial performance improvements than music intervention alone, consistent with previous research documenting its potent ergogenic properties in team-sport athletes. While not statistically superior to caffeine alone, the combined music–caffeine intervention produced the most significant absolute performance values across all measured parameters, suggesting potential additive effects between these interventions. These outcomes extend previous findings to team-sport contexts, indicating that ergogenic strategies targeting different physiological and psychological pathways may complement each other in performance enhancement scenarios. The significant improvements in jump height, agility, and repeated-sprint ability highlight the practical relevance of these strategies for sports requiring intermittent high-intensity efforts. These findings suggest that coaches working with female athletes might consider implementing a dual strategy approach during pre-competition preparation phases, particularly for morning competitions, when baseline arousal may be suboptimal. The absence of significant side effects further supports the practical application of these interventions. Future research might explore these effects in elite athletes and examine whether similar benefits occur across different menstrual cycle phases.

## Figures and Tables

**Table 1:** Prevalence of reported side effects immediately after exercise (day 1) and 24 h post-exercise (day 2). Data are expressed as percentages (%) of participants reporting the side effects. PLA: placebo; MUS: self-selected motivational music; CAF: caffeine supplementation; MUS + CAF: combination of music and caffeine.

	PLA		MUS		CAF		CAF + MUS	
Questionnaire	Day 1	Day 2	Day 1	Day 2	Day 1	Day 2	Day 1	Day 2
Muscle soreness	5.88	5.88	11.76	0	0	0	0	0
Increased urine output	0	0	0	5.88	0	0	0	5.88
Tachycardia	0	0	5.88	0	11.76	0	5.88	0
Anxiety or nervousness	0	0	0	0	0	0	5.88	0
Headache	5.88	5.88	0	0	11.76	0	11.76	0
Gastrointestinal problems	5.88	5.88	0	11.76	11.76	5.88	5.88	5.88
Insomnia	-	0	-	5.88	-	0	-	11.76
Increased vigor/activeness	5.88	0	11.76	0	5.88	0	23.52	0
Perception of performance improvement	17.64	-	17.64	-	17.64	-	23.52	-

**Table 2:** Accuracy of condition identification immediately after exercise. Data are expressed as percentages (%) of participants reporting the correct identification. PLA: placebo; MUS: self-selected motivational music; CAF: caffeine supplementation; MUS + CAF: combination of music and caffeine.

	PLA	MUS	CAF	CAF + MUS
Blinding	11.76	23.52	23.52	17.64

## Data Availability

The raw data supporting the conclusions of this article will be made available by the authors on request.

## Abbreviations

CAF	caffeine
CMJ	countermovement jump
MAT	modified agility *t*-test
MUS	music (self-selected motivational music)
PLA	placebo
RSA	repeated-sprint ability
RPE	rating of perceived exertion
SD	standard deviation
ANOVA	analysis of variance
PSQI	Pittsburgh Sleep Quality Index
FFQ	food frequency questionnaire
η^2^p	partial eta squared (effect size measure)
dB	decibels
BPM	beats per minute
BMI	body mass index
